# The history of Old World camelids in the light of molecular genetics

**DOI:** 10.1007/s11250-016-1032-7

**Published:** 2016-04-05

**Authors:** Pamela Anna Burger

**Affiliations:** Research Institute of Wildlife Ecology, Vetmeduni Vienna, Savoyenstrasse 1, 1160 Vienna, Austria

**Keywords:** *Camelus bactrianus*, *Camelus ferus*, *Camelus dromedarius*, Domestication, Genetic resource management, Genetic diversity

## Abstract

Old World camels have come into the focus as sustainable livestock species, unique in their morphological and physiological characteristics and capable of providing vital products even under extreme environmental conditions. The evolutionary history of dromedary and Bactrian camels traces back to the middle Eocene (around 40 million years ago, mya), when the ancestors of *Camelus* emerged on the North American continent. While the genetic status of the two domestic species has long been established, the wild two-humped camel has only recently been recognized as a separate species, *Camelus ferus*, based on molecular genetic data. The demographic history established from genome drafts of Old World camels shows the independent development of the three species over the last 100,000 years with severe bottlenecks occurring during the last glacial period and in the recent past. Ongoing studies involve the immune system, relevant production traits, and the global population structure and domestication of Old World camels. Based on the now available whole genome drafts, specific metabolic pathways have been described shedding new light on the camels’ ability to adapt to desert environments. These new data will also be at the origin for genome-wide association studies to link economically relevant phenotypes to genotypes and to conserve the diverse genetic resources in Old World camelids.

## Introduction

Without Old World camelids (Camelini), human survival and prosperity in the marginal agro-ecological zones of African, Arabian, and Asian (semi-) deserts would have been challenging. Since ancient times, camels were exploited as beast of burden, for riding, and as milk and meat source, and today, gain tremendous importance as sustainable livestock species with very specific features (e.g., immunogenes and milk composition). Camels facilitated the trading and cultural dialog between three continents by connecting the Arabian Peninsula with the Sahara and the Levant to the Far East and Asia, with northern Arabia at the crossroads.

Deep historical events like climate change or glacial periods as well as more recent anthropogenic interactions triggered demographic processes in Old World camelids, shaping the patterns of genetic diversity in modern populations. By examining this modern genetic diversity and its global distribution, it is possible to gain insight into past demographic events, which reflect the history of Old Word camelids. This review will give an overview about the evolutionary history of Old World camels and the molecular genetic studies performed over the past years, and summarize the standard molecular genetic methods that underlie these studies. In addition, recent advances in next-generation sequencing (NGS) technologies and their careful implementation in current and future camel research will be discussed.

## Methods for molecular genetic analysis

Depending on the objectives and size of the project, the choice of the marker is critical. The assessment of genetic diversity and population structure might be important if local or regional groups/breeds are investigated, while large-scale phylogenetic studies will be more interested in the global connectivity and demographic history of populations. Association studies between phenotypic traits and underlying genotypes will be necessary if marker-assisted breeding programs are anticipated or if adaptation to a specific environment is addressed.

### Genetic markers to investigate diversity and population structure

Molecular markers were previously applied in the camelid family for parentage control (Mariasegaram et al. 2002), as well as for the determination of the genetic variability between individual Bactrian camel (Chuluunbat et al. 2014; Silbermayr et al. 2010a; Jianlin et al. 2000, 2004) and dromedary breeds (Mburu et al. 2003), guanacos (Mate et al. 2005), llamas, and alpacas (Kadwell et al. 2001). Molecular evolutionary analysis was used to identify the ancestors of the llama and the alpaca (Kadwell et al. 2001; Stanley et al. 1994).

#### Maternal (mitochondrial) and paternal (Y-chromosomal) markers

The most commonly used polymorphic markers in camelid studies are single nucleotide polymorphisms (SNPs) or restriction length polymorphisms (RFLPs) studied in the mitochondrial DNA (mtDNA) reflecting the maternal inheritance (Silbermayr et al. 2010b; Ji et al. 2009; Jianlin et al. 1999). The genetic variation between Mongolian domestic and wild Bactrian camels showed 2.9 % nucleotide difference in the mitochondrial control regions (CR) of the two related species (Silbermayr et al. 2010b; Ji et al. 2009). In addition, mtDNA sequence analysis of ancient DNA (aDNA) proved to be crucial in resolving domestication processes in donkeys (Beja-Pereira et al. 2006), cattle (Troy et al. 2001), and dromedaries (Almathen et al., Ancient and modern DNA reveal dynamics of domestication and cross-continental dispersal of the dromedary, submitted to PNAS (2015-19508R)).

The Y-chromosome is much less variable within species, e.g., Y-SNPs (Lindgren et al. 2004), and Y-chromosome-linked microsatellite analysis detected high homology in different horse breeds (Wallner et al. 2003). Polymorphic Y-microsatellite markers in ruminants are currently only available for small ruminants (Meadows et al. 2006), yak (Xuebin et al. 2005), and cattle (Hanotte et al. 2000).

#### Nuclear autosomal markers

The main class of molecular markers used in molecular genetic studies in camelids is microsatellites, short repetitive motives (e.g., ACACAC…) in the nuclear DNA following the co-dominant inheritance (Schlötterer 2004). Autosomal microsatellite loci are important tools to measure population diversity (Chuluunbat et al. 2014; Silbermayr et al. 2010a; Jianlin et al. 2004), genetic variation, and genetic admixture among livestock breeds or between wild and domestic animal species (Bruford et al. 2003). A set of microsatellites established in New World camelids successfully amplified in the Camelini (Mariasegaram et al. 2002) and was applied to study the genetic distance between Mongolian and Chinese domestic Bactrian camels (Jianlin et al. 2004; Charruau 2012).

Nuclear SNPs are single base pair changes that occur approximately each 1000 bps in the mammalian genome. Large-scale nuclear SNP analyses have not been applied in Old World camelids so far. These markers are very useful alternatives to microsatellites and have been employed in many studies about genetic diversity and relevant phenotypic traits in livestock (Goddard and Hayes 2009).

### Population genetic and phylogenetic analyses in Old World camelids

The population genetic analyses in Old World camelids reviewed in this study were based on the comparisons of local breeds and addressed population genetic diversity and differentiation. If genetic diversity, population structure, and differentiation are the objectives of a study, the most important analytical steps should involve (1) evaluation of the raw data including confirming single haplotypes, testing for allelic dropout and possible null alleles, and checking for possible relatedness in the sample set to avoid a bias in the genetic diversity estimates, i.e., underestimation of heterozygosity and overestimation of the inbreeding coefficient (*F*_IS_) due to related individuals; (2) screening of population structure using Bayesian methods (Pritchard et al. 2000; Corander and Tang 2007) and/or principal component analysis (PCA); (3) estimation of principle population genetic and diversity parameters over all samples and between populations including Hardy-Weinberg equilibrium and heterozygosity (in nuclear markers), nucleotide and haplotype diversities (mtDNA and nuclear sequence data); (4) population differentiation in terms of population pairwise *F*_ST_ values and analysis of molecular variance (AMOVA); (5) phylogenetic and phylogeographic analyses constructing phylogenetic trees (Tamura et al. 2013) and haplotype networks (Bandelt et al. 1999); (6) inferences of demographic history, including pairwise mismatch distributions, calculations of neutrality estimators (e.g., Tajima’s D, Fu’s FS), and estimation of changes in the effective population size (*N*_e_) (Bouckaert et al. 2014; Beaumont 1999); and (7) individual hypothesis testing.

### Next-generation sequencing and genome-wide genotyping technologies: advantages and pitfalls

NGS usually refers to the sequencing of whole genomes using massive parallel sequencing technology. This method employs sequencing by synthesis (SBS; Ronaghi et al. 1998) with four fluorescently labeled nucleotides to sequence millions of single-molecule clusters on a flow cell in parallel, e.g., now offered by Illumina®. First draft whole genomes of the Old World camelids have recently been published (Fitak et al. 2015; Wu et al. 2014; Burger and Palmieri 2014; Jirimutu et al. 2012).

Genome-wide genotyping techniques involve high-density SNP chips or methods, which reduce genome complexity with restriction enzymes before massive parallel amplification, e.g., restriction site-associated DNA sequencing (RADseq; Andrews et al. 2016) and genotyping by sequencing (GBS; Hess et al. 2015). NGS and genotyping technologies applied to Old World camelids in the future might include whole-genome optical mapping for improved genome assemblies (Howe and Wood 2015) as well as SNP genotyping with different methods (RADseq, GBS). Furthermore, genome-wide association studies using either full genomes or high-density SNP chips might be performed similar to other livestock (Hayes et al. 2012; Goddard and Hayes 2009). Caveats of these new technologies include the necessity of high computational resources and advanced bioinformatics and statistical knowledge as well as a high sample number with well-defined phenotypes.

## Phylogenetic origin: three different species

The origin of the Old World camelids traces back to the Eocene (40–45 mya) when the first ancestors of the camelid family were found in North America. After splitting into New World (Lamini) and Old World (Camelini) camels, the latter migrated via the Bering land bridge to the eastern hemisphere (the Old World). The earliest camel remains in Asia date back to 5 mya (Kozhamkulova 1986). The divergence between one-humped and two-humped camels was estimated between 5 and 8 mya (Wu et al. 2014; Ji et al. 2009), contradicting previous assumptions of the wild Bactrian camel being the common direct ancestor of dromedary and Bactrian camels (Peters and von den Driesch 1997). Furthermore, an earlier observation of the dromedary embryo undergoing a two-humped developmental stage (Lombardini 1879) has been disproven by demonstrating the prenatal development of a single hump in Arabian camel fetuses (Kinne et al. 2010).

**Fig. 1:**
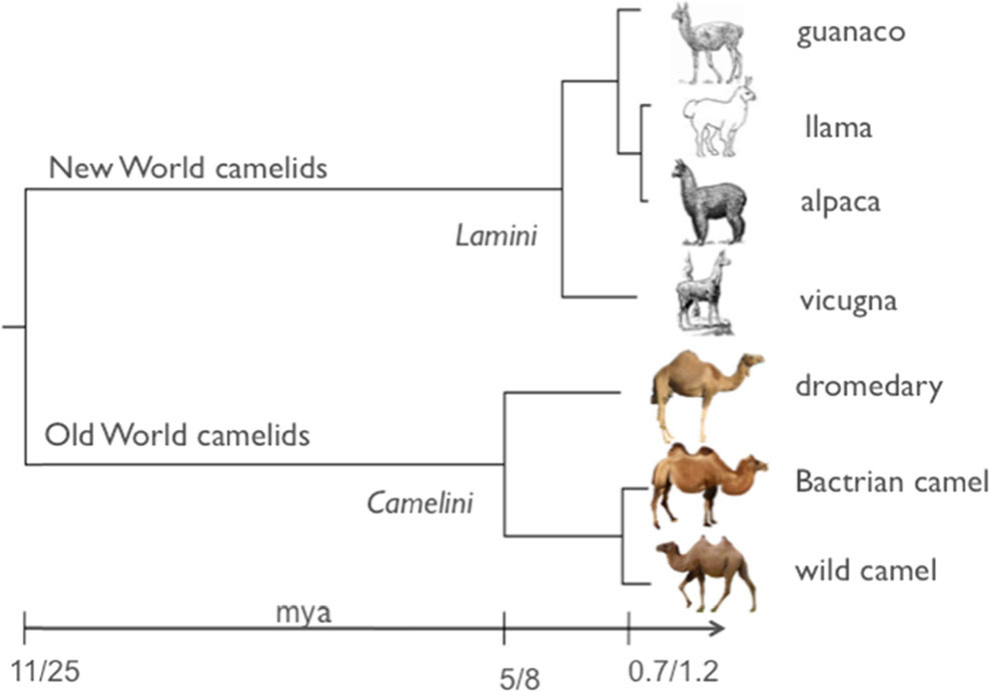
Maximum likelihood tree based on complete mitochondrial genomes of New and Old World camels

Molecular evolutionary studies estimate the split between Old and New World camels at 11 mya (Kadwell et al. 2001) to 25 mya (Ji et al. 2009). Within the Camelini, the divergence between dromedaries and Bactrian camels has been dated at 5 to 8 mya (Fig. 1) (Wu et al. 2014; Ji et al. 2009). Modern molecular genetic (Silbermayr et al. 2010b; Ji et al. 2009; Jianlin et al. 2004) and genomic (Jirimutu et al. 2012; Wu et al. 2014) studies confirm the presence of three extant Camelini species: *Camelus dromedarius*, *Camelus bactrianus*, and *Camelus ferus*. While the species status of both domestic species had been well established, it was heavily debated if the last wild two-humped camels in the Mongolian Gobi and the Chinese Taklimakan and Lop Nor deserts were feral or an evolutionary unique unit. Wild camels were first described by Przewalski in 1878, and the International Commission of Nomenclature (2003) fixed the first available specific name based on a wild population “*Camelus ferus*” to the wild camel (Gentry et al. 2004). Molecular genetic analysis of mitochondrial (Silbermayr et al. 2010b; Ji et al. 2009; Jianlin et al. 2004) and nuclear markers (Silbermayr and Burger 2012) demonstrated the divergence between wild and domestic Bactrian camels and estimated the time of separation between 0.7 and 1.5 mya in the Pleistocene, long before domestication (4000 – 6000 ya). This long-term divergence also excludes the wild two-humped camels as direct ancestors of modern domestic Bactrian camels.

**Fig. 2:**
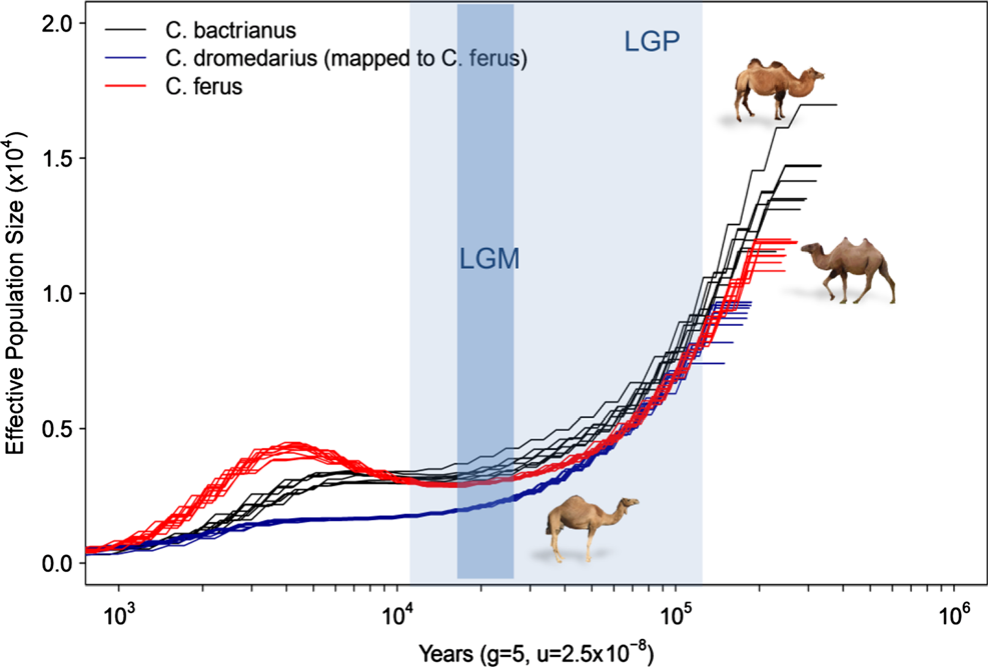
The historical demography of the three Old World camel species was examined using PSMC v0.6.4

The early independent demographic history of the three Old World camel species has now been reconstructed using whole-genome SNP data (Fitak 2014; Wu et al. 2014). The dataset consisted of whole-genome shotgun sequences from the three species of Old World camel (*C. dromedarius* (*n* = 9), *C. ferus* (*n* = 9), and *C. bactrianus* (*n* = 7)) mapped to the wild camel reference genome (Jirimutu et al. 2012) with approximately 15-fold coverage. To model the demographic history, the Pairwise Sequentially Markovian Coalescent model (PSMC; Li and Durbin 2011) was used with 100 bootstraps on the individual genomes masked for repetitive regions (Fitak 2014). PSMC infers *N*_e_ at a given time in the past from a single diploid individual using the rates of coalescence events across the genome. The number of coalescent events occurring more recently than 1000 ya is inadequate to accurately infer the demographic history. Figure 2 shows a maximum effective population size (*N*_e_) of 18,000 around 350,000 ya, with a sharp reduction of up to 70 % until the last glacial period (LGP; 100,000–20,000 ya). During the LGP, the *N*_e_ continued declining until it increased again in the Bactrian camel species and reached a plateau in the dromedaries after the end of the last glacial maximum (LGM; ∼20,000 ya). Similar patterns of demographic changes in large mammals based on climatic conditions during this period have been described (Orlando et al. 2013; Wu et al. 2014).

A second, more recent bottleneck starting around 4000–5000 ya further reduced the *N*_e_ in the Old World camels (Fig. 2). While this second bottleneck might be related with domestication scenarios of dromedary and Bactrian camels, in the case of the wild camel, it could reflect the beginning (and still ongoing) reduction of habitat and increased hunting activity connected with human population growth in Asia. Overall, a lower *N*_e_ in dromedaries than in Bactrian camels has been observed until the recent 3000–4000 ya (Fig. 2).

## Domestication

Domestic camels had an important role in the ancient civilizations of the Old World, and today, a number of rural and nomadic societies in North-eastern Africa and in Asia depend on these multipurpose animals in terms of transportation and milk, wool, meat, and dung production. The intensive trading and movements of camels around North Africa and the Arabian Peninsula are reflected in the mitochondrial (maternal) genetic profile of the global camel populations. A panmictic (globally unstructured) dromedary population ranging from North and East Africa to Pakistan and Australia was found (Charruau 2012). At the nuclear level, a more defined global structure was revealed, differentiating East African and South Arabian dromedaries from North African, North Arabian, and South Asian individuals, respectively (Almathen et al., Ancient and modern DNA reveal dynamics of domestication and cross-continental dispersal of the dromedary, submitted to PNAS (2015-19508R); Al-Mathen 2014; Charruau 2012; Mburu et al. 2003; Schulz et al. 2010). Wild dromedaries must have occurred in considerable numbers on the Arabian Peninsula in Holocene Times, as illustrated by the discovery of the second millennium BC hunting sites along the eastern coast yielding large amounts of bones from this species (von den Driesch and Obermaier 2007; Uerpmann and Uerpmann 2002). Zooarchaeological studies suggest that domestication took place in the coastal southeast Arabian Peninsula (Uerpmann and Uerpman 2002, 2012; von den Driesch and Obermaier 2007; Grigson 2012, 2014). Ongoing genetic studies combining modern and ancient DNA confirm this region as one possible place of domestication and suggest a single domestication scenario with recurrent introgression from wild dromedaries into the early-domestic stock (Almathen et al., Ancient and modern DNA reveal dynamics of domestication and cross-continental dispersal of the dromedary, submitted to PNAS (2015-19508R)).

The phylogeographic analysis of modern domestic Bactrian camels from Mongolia, Russia, Kazakhstan, and China revealed that mitochondrial haplotypes are shared between animals and countries (Charruau 2012). Moreover, a palaeogenetic analysis of 12 Bactrian camel bones from Late Bronze and Early Iron Age sites in Uzbekistan and Siberia showed the same mitochondrial haplotypes as described in modern domestic Bactrian camels, which were genetically distinct from wild Bactrian camels. This reveals that the current Mongolian wild camels are neither the descendants of Late Bronze and Early Iron Age nor the ancestors of modern domestic Bactrian camels. The high homogeneity between these Bronze/Iron Age samples and modern domesticated camels from China and Mongolia led to the conclusion that there was a single domestication for Bactrian camels (Trinks et al. 2012). Until today, the wild relatives of the domestic Bactrian camel still exist in small numbers in Mongolia and China whereas wild dromedaries have become extinct.

## Hybridization

Anthropogenic hybridization between domestic animals and their wild relatives has occurred since the early days of animal breeding. In Old World camels, we find two directions, (i) hybridization between Bactrian camels and dromedaries, which facilitates strong and robust individuals; and (ii) introgression (gene flow from one pool into the other over more generations) from domestic Bactrian camels into wild two-humped camels, and survival of the wild species classified as “critically endangered” by the International Union for Conservation of Nature (IUCN).

### The encounter of dromedary and Bactrian camels

Hybridization between Bactrian camels (*C. bactrianus*) and dromedaries (*C. dromedarius*) was associated with the transportation of goods along multiple routes of the Silk Road. This practice intended to produce animals with the robustness of the Bactrian camel, the endurance of dromedary, and the ability to tolerate sharply contrasting climatic conditions (Wilson 1984). Today, hybridization facilitates improved milk and wool yield in hybrid *Tulu* or *Nar* camels from Middle Eastern and Central Asian countries. Commonly, two hybridizing methods are recognized, *Kurt*-*nar* (dromedary female × Bactrian male) and *Kez*-*nar* (Bactrian female × dromedary male) followed by *F*_1_-backcrossing with either dromedary for increased milk productivity or Bactrian camel for wool and cold resistance (Faye and Konuspayeva 2012). This heterosis or hybrid vigor effect arises from allelic interactions between parental genomes, potentially leading to increased growth, productivity, and fitness of the hybrids. *F*_2_ hybrids (*F*_1_ × *F*_1_) in Old World camels are usually not favored because of a difficult character and weak progeny performance (Faye and Konuspayeva 2012). A SNP marker set has been developed to identify hybrids and their backcrosses in animals with a cryptic ancestry, or in ancient samples (Ruiz et al. 2015).

### Hybridization and introgression between wild and domestic Bactrian camels: a matter of conservation

**Fig. 3:**
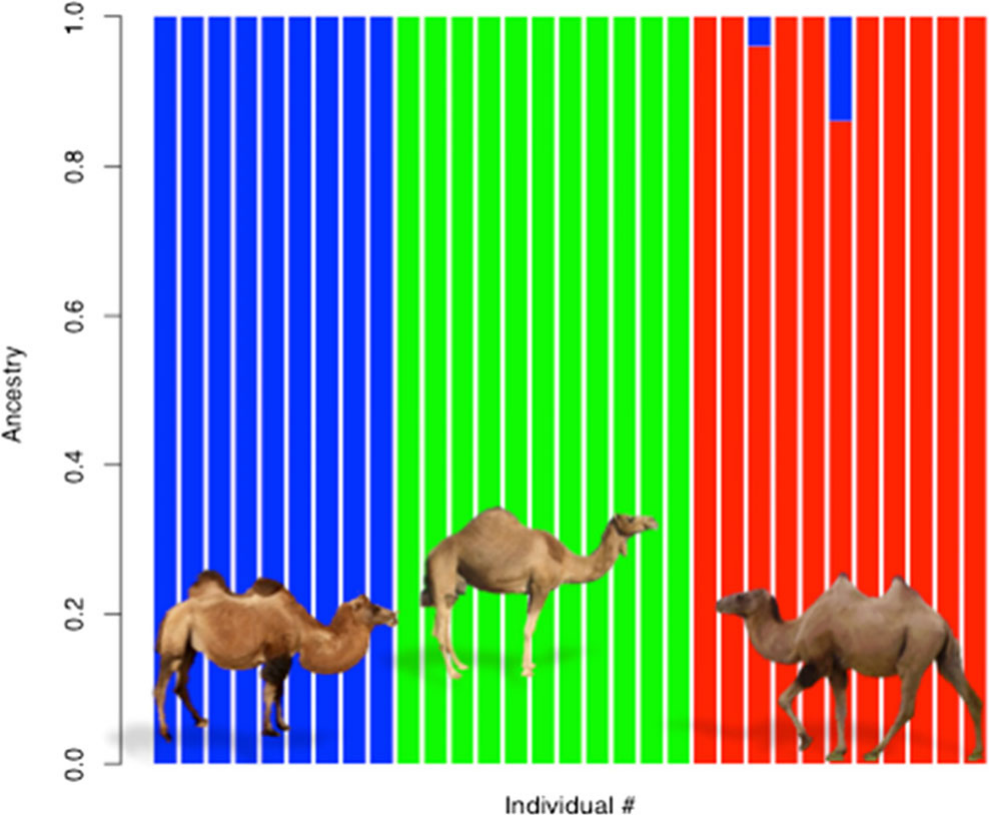
Admixture analysis using whole-genome SNP data of dromedary (*green*), Bactrian (*blue*), and wild (*red*) camels. Each *bar* represents the genomic ancestry of a single individual; *mixed colors* indicate a mixed ancestry between two species

The hybridization between domestic Bactrian and wild camels is jeopardizing a long process of evolution as it threatens the gene pool of the wild species (Lei et al. 2012; Yadamsuren et al. 2012). With nuclear and mitochondrial markers, different levels of admixture between domestic and wild camels were detected in the Mongolian wild camel population (Charruau 2012; Silbermayr et al. 2010b). This has also been confirmed using whole-genome SNPs as two supposedly pure wild camels showed introgression from domestic camels (Fig. 3; Fitak et al., unpublished data). “Preserving the genetic integrity of the wild camel” was included as a conservation priority to the Mongolian National Conservation Strategy for the wild camel and its desert habitat (2012).

## Ongoing and future research

The genomic resource “Old World camels” has come into focus in the last decade, with a number of scientific groups working in different fields.

### Adaptation to desert environment and selection during domestication

Genes involved in energy metabolism and stress response were described to show accelerated evolutionary rates as compared to New World camels or cattle. Similarly, rapid evolution of genes underlying pathways for salt metabolism and duplications of genes, which play critical roles in sodium reabsorption and water balance in the kidney, were defined to be related to an adaptation to desert environment (Wu et al. 2014; Jirimutu et al. 2012). Comparative analyses between domestic dromedary and Bactrian camels versus wild camels aim to detect signatures of selection during domestication in Old World camels (Fitak et al., unpublished data).

### Immunogenetics

The camel immunogenome has been studied so far only to limited extent (e.g., Griffin et al. 2014; Muyldermans et al. 2009). Ongoing studies target the major histocompatibility complex (MHC), a set of immune response (IR) genes playing a crucial role in host-pathogen interactions, to characterize the general organization and molecular evolution of this region in Old World camels (Plasil et al. 2016). Other IR genes, like the T cell receptor, have evolved in the dromedary by mutation in the gamma (TRG) and delta (TRD) genes (Ciccarese et al. 2014). The genomic structure and gene content of these genes are currently investigated (Antonacci et al. 2015).

### Genetic analyses of production traits

The encoding genes for β-casein (Pauciullo et al. 2014) and κ-casein (Pauciullo et al. 2013), two major abundant proteins in camel milk, have been characterized in dromedaries and partially in Bactrian camels. The studies confirm the potential protective role of the camel milk for the human nutrition. The comprehensive characterization of the sequence variability at the MSTN locus coding for myostatin, a negative regulator of skeletal muscle responsible in several livestock species for increased skeletal-muscle mass, in Tunisian dromedaries has been initiated (Muzzachi et al. 2015).

### New challenges: genome-wide association studies and genomic selection

The identification of genotypes underlying economically relevant phenotypic traits using genome-wide association studies (GWAS) has been applied in other domestic species for a long time (Goddard and Hayes 2009). Selective breeding has tremendously increased market weight in chicken and pigs, and milk production in cattle (Hayes et al. 2012). The trade-off of this intensive selection, however, is loss of genetic diversity, inbreeding and interconnected problems of decreased fertility, and a reduced potential for adaptation to environmental and pathogenic changes. The challenges in this field are numerous, and nowadays many breeding programs shift from only production to include health and fitness traits, like fertility (Hayes et al. 2012).

Genomic selection refers to the application of genome-wide markers to predict the breeding value of selection candidates (Meuwissen et al. 2001). The major improvement over earlier methods of marker-assisted selection, which relied on a small number of causal variants, is the association of complex traits with hundreds or thousands of polymorphisms, each with small effects (Goddard and Hayes 2009). The computation to predict breeding values from SNP genotypes must be estimated from a large sample of animals (around 1000, depending on *N*_e_ and linkage disequilibrium in the population), termed the reference population, which have been phenotyped (measured for certain traits) and SNP genotyped. Breeding values for selection candidates solely based on their genotypes can then be calculated with a prediction equation. The candidates are ranked on these estimated breeding values and the best ones are selected to breed the next generation. The advantage of genomic selection is that based on their genomic prediction, individuals can be selected accurately much earlier in life, which results in a reduction of the generation interval and an increase in the rate of genetic gain (Hayes et al. 2012).

In Old World camels, the challenge for future sustainable breeding programs will be to balance the intensification of production traits with the preservation of genetic diversity and local adaptation. The development of high-density SNP chips or potential whole-genome sequencing projects similar to the 1000 bulls genome project (www.1000bullgenomes.com) as a basis for genomic selection need to go along with a thorough and standardized recording of production traits, pedigree, and progeny performance testing. Potential genomic selection projects in camels need to be preceded and complemented by traditional breeding programs based on pedigrees and phenotypes.

### Conservation of genetic resources in wild and domestic camels

The level of genetic variation and as such the ability of a population to evolve and respond to artificial and natural selection is positively associated with the effective population size *N*_e_ (Falconer and Mackay 1996). In natural (wild) populations, there are well-known cases of inbreeding and decreased adaptation potential due to reduced genetic variation (Kristensen et al. 2015). Similar to natural populations, inbreeding depression has been observed in domestic species with a small *N*_e_, decreasing fertility and milk yield in cattle (Pryce et al. 2002), and growth rates in sheep (Pedrosa et al. 2010), while increasing diseases (e.g., mastitis; Sørensen et al. 2006). We still record average levels of genetic diversity (nuclear heterozygosity and nucleotide diversity) in domestic Bactrian camels (Chuluunbat et al. 2014) and dromedaries (Almathen 2014; Charruau 2012). However, in wild camels, reduced genetic variation and smaller census size as well as lower *N*_e_ have been observed (Charruau 2012; Yadamsuren et al. 2012). Thoughtfully applied genomic tools can contribute to a genetic resource management through monitoring and preserving genetic uniqueness (i.e., *C. ferus*) and genetic diversity, while improving vitally important production traits in the amazing and precious Old World camels.
